# Strength Tests and Mechanism of Composite Stabilized Lightweight Soil Using Dredged Sludge

**DOI:** 10.3390/ma18020348

**Published:** 2025-01-14

**Authors:** Qizhi Hu, Zitian Li, Qiang Ma, Junjie Li, Wei Yao

**Affiliations:** 1 School of Civil Engineering, Architecture and Environment, Hubei University of Technology, Wuhan 430068, China; hqz0716@163.com (Q.H.); maqiang927@163.com (Q.M.); ljj72700829@163.com (J.L.); 102200838@hbut.edu.cn (W.Y.); 2 Bridge Safety Monitoring Technology and Equipment Engineering Technology Research Center, Hubei University of Technology, Wuhan 430068, China

**Keywords:** roadbed engineering, dredged sludge, expanded polystyrene particles, lightweight soil, unconfined compressive strength, microstructural mechanism

## Abstract

To achieve resourceful utilization of dredged sludge, lightweight treatment was performed on sludge from Xunsi River in Wuhan using fly ash, cement, and expanded polystyrene (EPS) particles. Density tests and unconfined compressive strength (UCS) tests were conducted on the composite stabilized sludge lightweight soil to determine the optimal mix ratio for high-quality roadbed fill material with low self-weight and high strength. Subsequently, microstructural tests, including X-ray diffraction (XRD) and scanning electron microscopy (SEM), were conducted. The Particle (Pore) and Crack Analysis System (PCAS) was used to analyze the SEM images, investigating the cement–fly ash composite stabilization mechanism. The experimental results showed that the optimal lightweight treatment was achieved with an EPS content of 80% (by volume ratio to dry soil), cement content of 7.5% (by mass ratio to dry soil), and fly ash content of 5% (by mass ratio to dry soil). The density of the optimized lightweight soil was 1.04 g/cm^3^, a reduction of 28.27% compared to the density of raw sludge soil (1.45 g/cm^3^). The UCS increased significantly from 110 kPa for raw sludge soil to 551 kPa. The addition of fly ash enhanced the hydration and secondary hydration reactions between cement and sludge, generating more calcium silicate hydrate (C-S-H) gel, which filled the larger pores between the EPS particles and soil particles, as well as those between the soil particles themselves, making the structure denser. Compared to single cement stabilization, composite stabilization resulted in a lower content of expansive ettringite crystals, a more uniform pore distribution, fewer pores, and a lower surface porosity ratio. These research findings can provide theoretical support and practical references for the lightweight treatment of dredged sludge in the Yangtze River Basin of Central China.

## 1. Introduction

With the rapid urbanization process in China, infrastructure construction in coastal and riverside areas frequently encounters the challenge of dealing with dredged sludge foundations. Dredged sludge is characterized by high water content, low strength, high sensitivity, and high fluidity [1,2], making it unsuitable for direct use in practical civil engineering. Currently, large-scale treatment of dredged sludge foundations in China primarily relies on replacement and drainage consolidation [3]. However, the processes of consolidation, transportation, and disposal of a massive volume of sludge result in severe resource wastage and environmental pollution. With the introduction of concepts such as environmental protection, conservation of soil and water resources and recycling of solid waste, traditional treatment methods for dredged sludge foundations could not meet the requirements of modern infrastructure construction [4].

To solve the problems such as poor stability, uneven settlement, and low bearing capacity of dredged sludge foundations, lightweight treatment technology has been introduced. Lightweight treatment refers to mixing lightweight materials, curing agents, and water into the raw soil to form lightweight soil after sufficient curing. Lightweight materials may include EPS particles [5], foam [6], and waste rubber particles [7], while curing agents may consist of cement, lime, and other cementitious materials. Lightweight construction materials, such as foamed concrete and EPS particle lightweight soil, possess advantages such as low density, thermal insulation, soundproofing, and earthquake resistance. These materials have been widely applied in roadbed engineering, foundation treatment, excavation backfilling, and building construction fields [8,9,10].

Traditional research on lightweight soil focuses on its preparation processes, mechanical properties, microstructure, and constitutive models [11,12,13,14]. This technology enables the large-scale treatment of dredged sludge into lightweight soil that can be reused as backfill on-site, thus allowing dredged sludge foundations to meet engineering requirements. Jifengling studied the progressive failure process of lightweight dredged sludge soil under isotropic and uniaxial compression, describing the development of cracks in lightweight soil from both macro and micro perspectives [15,16]. Liu Zengxiang conducted density and permeability tests and performed a sensitivity analysis on the density and permeability of lightweight sludge soil using an orthogonal data processing method. In the orthogonal data analysis, the within-group range of the impact of lightweight material content on density was 0.23, and the within-group range of the impact of water content on permeability was 2.84, both higher than those of other influencing factors. This led to the conclusion that the main controlling factors for the density and permeability of lightweight sludge soil in engineering applications are the content of lightweight materials and water content [17]. Zhang Panpan used ABAQUS software (version 6.14-3) to conduct finite element simulation of lightweight sludge soil subgrade and validated the simulation results using a one-dimensional settlement test [18].

At present, cement is the most widely used solidification material in the lightweight treatment of sludge. However, cement production consumes a significant amount of energy and generates industrial waste and harmful gases, causing irreversible environmental damage. Fly ash is one of the main solid wastes discharged from coal-fired power plants, with its chemical composition primarily consisting of SiO_2_, Al_2_O_3_, and Fe_2_O_3_. As a cementitious material, fly ash has potential pozzolanic activity, and when mixed with cement, it can effectively enhance the strength and ductility of the cement [19]. In this study, fly ash was used to partially replace cement to produce a fly ash–cement composite solidifying agent, and expanded polystyrene (EPS) particles were used as lightweight material to perform treatment of the sludge. This study conducted density tests and unconfined compressive strength tests on composite solidified lightweight soil made from dredged sludge, analyzing the effects of the content of each component on the density and unconfined compressive strength of the composite solidified lightweight soil. Additionally, combined with XRD and SEM tests, a PCAS system was used to analyze the pore quantity, distribution characteristics, and pore size of the composite solidified lightweight soil made from dredged sludge, investigating its composite solidification mechanism from a microscopic perspective.

## 2. Materials and Methods

### 2.1. Text Materials

**Figure 1:**
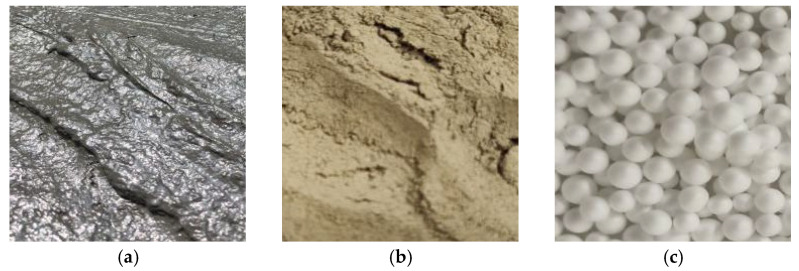
Test materials: (**a**) dredged silt, (**b**) dried dredged silt [20], (**c**) EPS particles.

This experiment utilized dredged sludge as the base soil material, combined with fly ash and cement as curing agents and expanded polystyrene (EPS) particles as lightweight additives (Figure 1). The basic physical properties obtained from the natural moisture content test and compaction test are shown in Table 1.

The raw sludge used in the experiment was collected from Xunsi River, a tributary of the Yangtze River in Wuhan, Hubei Province. The riverbed sludge was in a saturated state, dark gray in color with occasional black inclusions. It had a smooth surface, good plasticity, and an unpleasant fishy odor. The basic physical properties of the sludge were obtained through indoor testing, as shown in Table 1. Before use, the sludge was oven-dried, crushed, and sieved through a 2 mm mesh.

The fly ash used in the experiment was classified as standard Grade II fly ash, with its chemical composition provided by the manufacturer (Henan Hengyuan New Material Co., Ltd., Henan, China), as listed in Table 2. The cement used was 42.5# Portland cement produced in Hubei. The EPS particles used were spherical, with a particle size ranging from 1 to 2 mm. Through testing, the density of the EPS particles was measured to be 0.025 g/cm^3^, and the bulk density was determined to be 0.017 g/cm^3^.

### 2.2. Test Scheme

The EPS content was determined based on its volume ratio, while the fly ash and other components were measured by mass ratio. In roadbed engineering, the typical cement content in cement-stabilized soil is around 5% [21]. For the special case of EPS-reinforced lightweight dredged sludge soil, with a higher cement content (≥10%), the high cost of construction materials would make large-scale treatment difficult and contradict the concept of green and sustainable development. Therefore, the cement content was fixed at 7.5%. Fly ash was partially used to replace cement to reduce cement consumption while ensuring that the composite stabilized lightweight soil met high strength standards.

The EPS content (a_e_) was set at 60%, 80%, and 100%, and fly ash (a_c_) was added in proportions of 0%, 2.5%, 5%, 7.5%, and 10%. Samples were cured for 28 days, and density and unconfined compressive strength (UCS) tests were conducted (Table 3) to identify an optimal mix design that combines low self-weight, high strength, and environmental sustainability.

### 2.3. Specimen Preparation

The sample preparation method is as follows: A specific amount of dredged sludge was mixed with predetermined quantities of cement and fly ash in a mixer, following the experimental scheme. EPS beads and water were then added in proportion and mixed thoroughly using the mixer. Mixing was stopped once a homogeneous mixture was achieved.

The prepared mixture was compacted into cylindrical molds with a diameter of 39.1 mm and a height of 80 mm. The compaction was conducted in four layers using a three-part split mold. Each layer was compacted with 27 blows, and a diamond-pattern scraper was used between layers to create rough surfaces that enhanced interlayer bonding. Finally, the surface of the specimen was leveled with a spatula. A total of 30 groups of samples were prepared for unconfined compressive strength tests and density tests, with each group consisting of three parallel samples. The samples were cylindrical with a diameter of 39.1 mm and a height of 80 mm.

After preparation, the specimens, still inside the molds, were stored in a standard curing chamber maintained at a constant temperature of 20 ± 2 °C. After 24 h, once the cement and fly ash had initially set, the molds were carefully removed. The demolded specimens were then wrapped in plastic film and returned to the curing chamber for continued curing until reaching the target age of 28 days.

**Figure 2:**
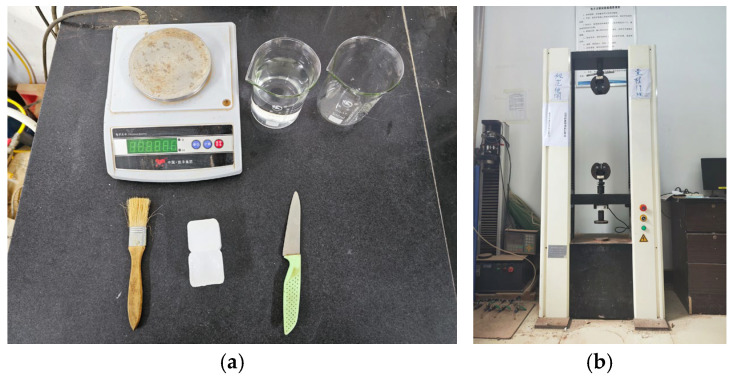
Test Instruments: (**a**) density test, (**b**) UCS test.

Density is an important indicator for evaluating the characteristics of lightweight soil and effectively reflects its lightweight properties. The density test was conducted using the wax-sealing method, following the standard geotechnical testing method (GB/T 50123-2019) [22]: ① Cut a soil block with a volume no less than 30 cm^3^. Use a soil-cutting knife to remove any loose soil and sharp edges from the block’s surface. ② Tie a thin thread around the soil block and measure the combined mass of the soil block and thread, denoted as *m*_1_. ③ Melt the wax by placing wax blocks in a beaker heated in a water bath. Hold the thread and fully immerse the soil block into the molten wax, then remove it immediately. ④ Inspect for air bubbles around the wax coating. Use a heated needle to puncture any air bubbles, and use a brush dipped in wax to fill any gaps. Once the wax has cooled and fully sealed the soil block, measure the mass of the wax-sealed sample, denoted as *m*_2_. ⑤ Submerge the wax-sealed sample in pure water using a stand, ensuring it is fully immersed. Record the mass of the wax-sealed sample in water, denoted as *m*_3_. ⑥ Remove the wax-sealed sample from the water, wipe it dry with a towel, and reweigh it to confirm that no water has penetrated the wax seal. The test instruments are shown in Figure 2a, and the soil density was calculated using Equation (1). Each sample underwent three parallel tests, and the final density result was taken as the average of the three test values.

(1) $$\rho_{s}=\frac{m_{1}}{\frac{m_{2}-m_{3}}{\rho_{w}}-\frac{m_{2}-m_{1}}{\rho_{p}}}$$

where *m*_1_ is the mass of the soil block (g), *m*_2_ is the mass of the paraffin wax-sealed soil block (g), *m*_3_ is the mass of the paraffin wax-sealed soil block in water (g), *ρ_s_* is the density of the soil block (g/cm^3^), *ρ_w_* is the density of water (g/cm^3^), and *ρ_p_* is the density of paraffin wax (g/cm^3^).

The unconfined compressive strength (UCS) test was performed using a WDW-10E micro-controlled electronic universal testing machine (Chenda Testing Machine Manufacturing Co., Ltd. (Jinan, China)). The test apparatus is shown in Figure 2b. During the test, the loading rate was controlled at 1 mm/min, and the test was terminated when the axial strain reached 6%. The stress–strain curve was plotted with axial strain as the horizontal axis and axial stress as the vertical axis. The UCS of the specimen was determined by the peak axial stress. The UCS of the specimen was calculated using Equation (2).

(2) $$q_{u}=\frac{P}{A}$$

where *q_u_* is the specimen’s unconfined compressive strength (MPa), *P* is the maximum failure load (N), and *A* is the cross-sectional area (mm^2^).

## 3. Results

### 3.1. Density Tests and Influencing Factors

Density is a key physical property of lightweight soil used as a filling material. To explore the impact of different mixing ratios on lightweight soil density, density tests were conducted based on the experimental plan outlined in Table 3. The cement content was held constant at 7.5%, producing 15 groups of specimens with varying EPS and fly ash content. Three specimens were prepared for each group with the same mixing ratio, and the average density of the three was calculated. The wax-sealing method was employed for the density measurements.

#### 3.1.1. Effect of EPS Content on Density

**Figure 3:**
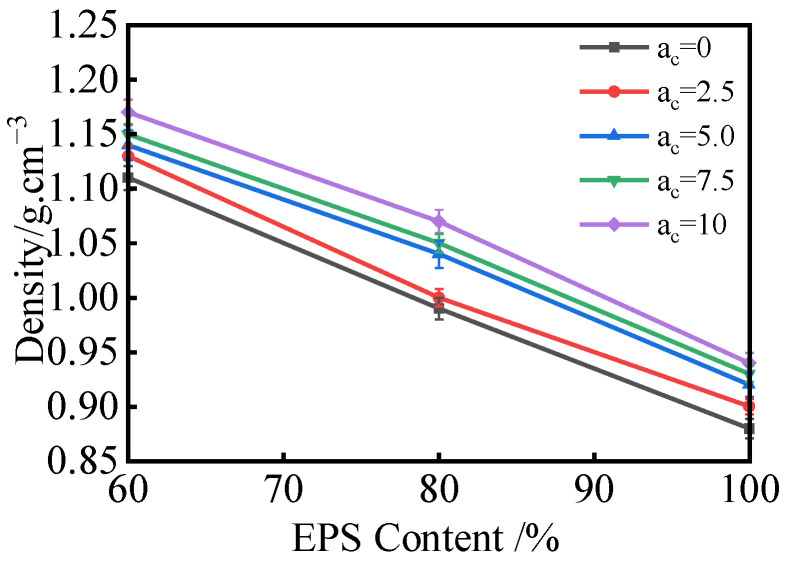
Density Variation Curve Under Different EPS Contents.

Figure 3 illustrates the changes in lightweight soil density at different EPS content levels. According to Figure 3, the density of the five specimen groups ranged between 0.90 g/cm^3^ and 1.17 g/cm^3^, with intergroup density differences spanning 0.02 g/cm^3^ to 0.15 g/cm^3^ and intragroup differences from 0.20 g/cm^3^ to 0.23 g/cm^3^. These results indicate that the EPS content significantly influences the density of lightweight soil.

#### 3.1.2. Effect of Fly Ash Content on Density

**Figure 4:**
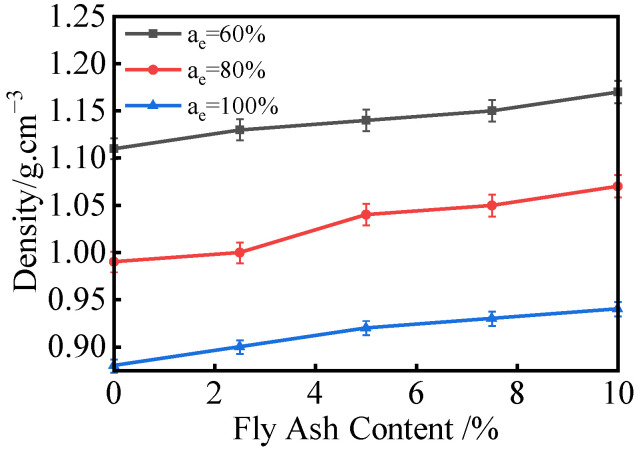
Density Variation Curve Under Different Fly Ash Contents.

The variation in lightweight soil density with fly ash content is shown in Figure 4. The results indicate that fly ash content has a relatively minor effect on the density of lightweight soil. Since the density of fly ash is higher than that of the composite stabilized lightweight soil, the density of the specimens slightly increases with the increase in fly ash content.

### 3.2. Unconfined Compressive Strength Test and Results Analysis

UCS is also a key physical property of lightweight soil used as a filling material. To explore the impact of different mixing ratios on lightweight soil UCS, tests were conducted based on the experimental plan outlined in Table 3. The cement content was held at 7.5%, producing 15 groups of specimens with varying EPS and fly ash content. Three specimens were prepared for each group with the same mixing ratio, and the average UCS of the three was calculated.

#### 3.2.1. Stress–Strain Characteristics of Composite Stabilized Lightweight Soil

**Figure 5:**
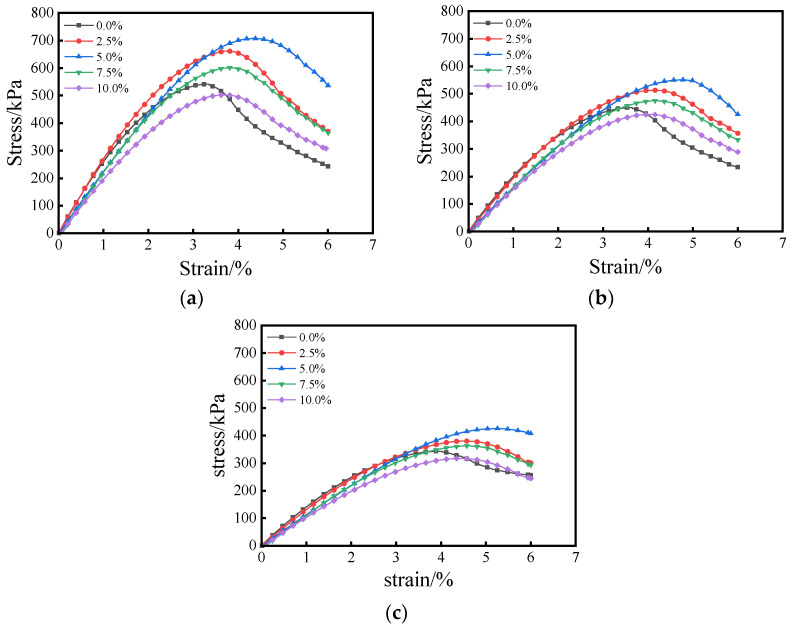
Stress–Strain Curve for Different Fly Ash Contents at Different EPS Contents: (**a**) EPS content 60%, (**b**) EPS content 80%, (**c**) EPS content 100%.

The stress–strain curves of composite stabilized lightweight soil are shown in Figure 5. The uniaxial compression deformation process of the composite stabilized lightweight soil can be divided into three stages: compaction, elastic deformation, and yielding. When the axial strain is within 2.5%, the stress–strain curve appears approximately linear with a high slope, and stress increases rapidly, indicating that the composite stabilized lightweight soil exhibits elastic deformation at the initial stage of strain. When the axial strain is between 2.5% and the peak stress, the increase in axial stress becomes more gradual. After reaching the peak stress, the stress begins to decline.

At the same fly ash content, the peak stress of the composite stabilized lightweight soil decreases as the EPS content increases. Additionally, the failure strain at peak stress increases, the stress drop becomes slower, and the material exhibits more pronounced softening deformation characteristics. Conversely, at a constant EPS content, as the fly ash content increases, the peak stress of the composite stabilized lightweight soil increases, the failure strain at peak stress decreases, the stress drop becomes more rapid, and the material demonstrates more apparent hardening deformation characteristics.

A comprehensive analysis of the stress–strain curves for composite stabilized lightweight soils with different mix ratios (Figure 5) reveals that the overall stress–strain curves are parabolic in shape, and the variation trends are consistent across all specimens. The UCS of untreated raw sludge is significantly lower than that of the improved composite stabilized lightweight soil.

#### 3.2.2. Effect of EPS Content on Unconfined Compressive Strength

**Figure 6:**
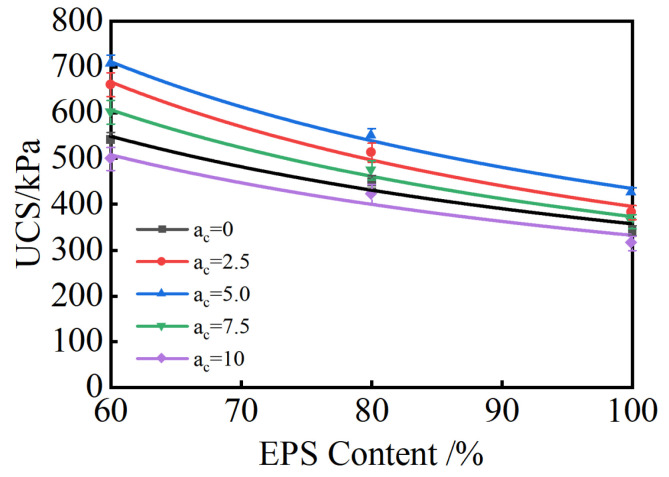
Effect of EPS Content on Unconfined Compressive Strength Curve.

Figure 6 illustrates the effect of EPS content on the unconfined compressive strength of lightweight soil. The analysis (Figure 6) shows that the UCS decreases as the EPS content increases. When the EPS content increases from 60% to 100%, the UCS of the composite stabilized lightweight soil decreases from 499–707 kPa to 315–425 kPa, with an average reduction of 38.6%.

When the soil body is subjected to external force, the primary load-bearing components are the skeleton formed by EPS particles and soil particles treated with the cement–fly ash stabilizer. When the EPS content is high, the EPS particles occupy a large volume fraction of the composite solidified lightweight soil, causing soil particles to exist only in the gaps between EPS particles. This disrupts the skeleton formed by the EPS particles and soil particles, preventing the stabilizer’s cementitious effect from being fully utilized, which results in lower strength of the composite lightweight soil.

Appropriate incorporation of EPS particles can improve the brittleness of the composite solidified lightweight soil, reduce its density, and minimize strength loss without disrupting the cementitious effect of cement and fly ash. The samples exhibited shear failure at the point of destruction, with a distinct shear plane visible, and no EPS particles were found to be sheared or damaged. When the EPS particle content was relatively high, numerous cracks were observed on the surface of the samples during failure.

#### 3.2.3. Effect of Fly Ash Content on Unconfined Compressive Strength

**Figure 7:**
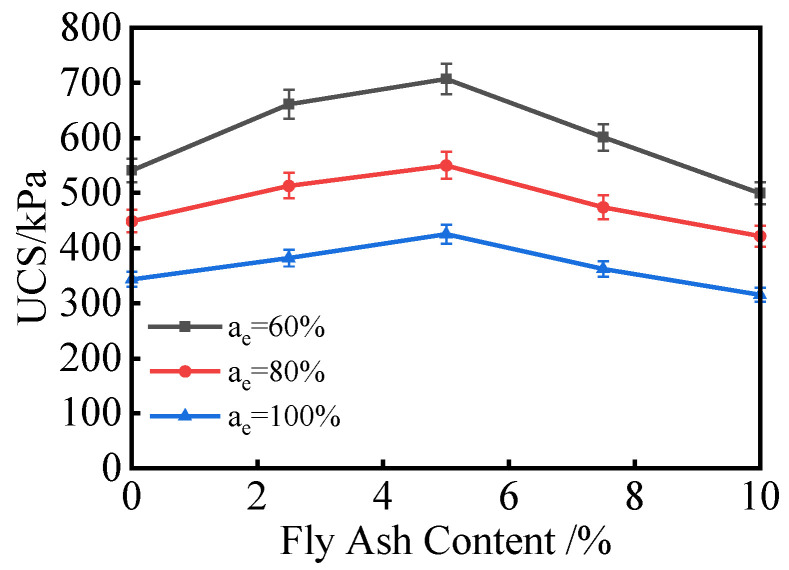
Effect of Fly Ash Content on Unconfined Compressive Strength Curve.

Many studies have shown that due to its relatively low strength, fly ash cannot be used as a standalone stabilizer for sludge-based materials and needs to be combined with materials such as lime, gypsum, or carbide slag [23]. Figure 7 shows the effect of fly ash content on the unconfined compressive strength (UCS) of composite stabilized lightweight soil. The analysis (Figure 7) shows that at a constant EPS content, the UCS initially increases and then decreases as the fly ash content increases, reaching its peak at a fly ash content of 5%.

When the fly ash content is 0%, only the hydration reaction of cement occurs. However, due to the relatively low cement content, the UCS of the composite stabilized lightweight soil is also low. At a fly ash content of 5%, the curing agent in the lightweight soil becomes a fly ash–cement composite, where the fly ash reacts with the hydration products of cement in a secondary hydration process, enhancing the soil’s strength. When the fly ash content reaches 8–10%, excessive fly ash is incorporated, leaving a large amount of unreacted fly ash after the secondary hydration process. Additionally, the inherent low strength of fly ash hydration reactions, combined with issues such as particle agglomeration and incomplete reactions, leads to a reduction in lightweight soil strength.

This indicates that the incorporation of fly ash can enhance the UCS of composite stabilized lightweight soil. However, indiscriminately increasing the fly ash content will not continuously improve the strength of the soil.

### 3.3. X-Ray Diffraction Test (XRD)

**Figure 8:**
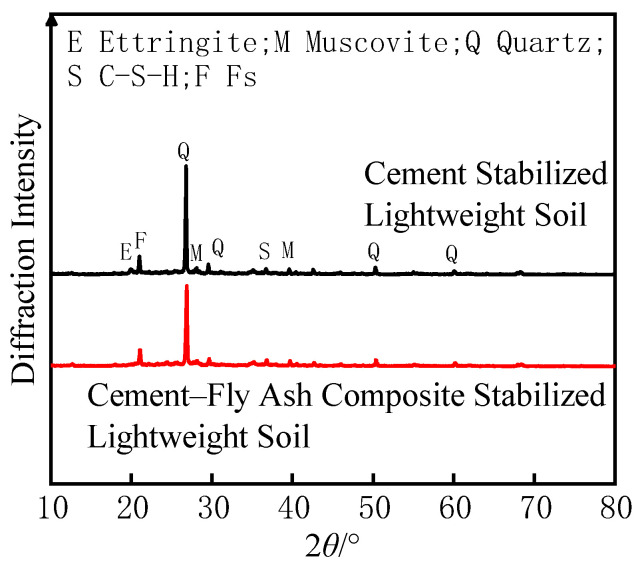
XRD Pattern of Stabilized Soil.

The XRD patterns of cement stabilized soil and cement–fly ash composite stabilized soil are shown in Figure 8. The hydration reaction between cement and dredged sludge produces calcium silicate hydrate (C-S-H), while sulfates and chlorides in the sludge promote the formation of ettringite (AFt) and hydrated calcium aluminate chloride (FS) [24]. Ettringite, a needle-like crystal, tends to align directionally or form crystal agglomerates in cement-stabilized soil, which can lead to a reduction in soil strength.

Compared to cement-stabilized soil, the diffraction intensities of quartz, muscovite, and other minerals in cement–fly ash composite stabilized soil are lower, indicating that more natural minerals participated in the hydration reactions. Although diffraction peaks corresponding to ettringite are still present in cement–fly ash composite stabilized soil, their intensity is significantly reduced.

### 3.4. Scanning Electron Microscopy Test (SEM)

**Figure 9:**
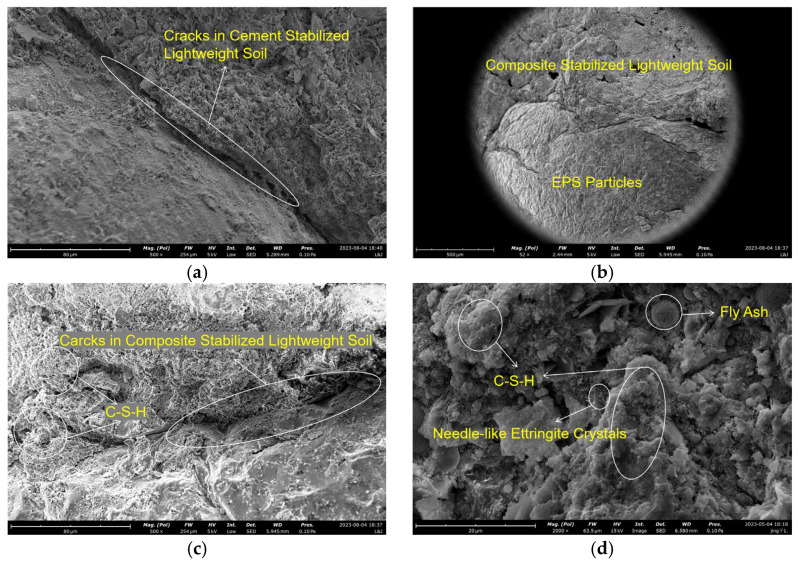
SEM Images of Lightweight Soil: (**a**) cement stabilized (500 times), (**b**) composite stabilized (50 times), (**c**) composite stabilized (500 times), (**d**) composite stabilized (2000 times).

A SEM image of lightweight soil with cement as the sole curing agent is shown in Figure 9a. It reveals large cracks in the stabilized soil, with numerous needle-shaped ettringite crystals and crystal agglomerates observed between the EPS particles and stabilized soil, as well as between soil particles. This phenomenon is a common issue in cement-based materials and a primary cause of intrinsic cracks and strength reduction in such materials.

Figure 9b presents a SEM image of composite stabilized lightweight soil. In this image, the EPS beads are largely encapsulated by the surrounding stabilized soil. This phenomenon allows the lightweight soil to be simplified into an EPS-bead and stabilized soil dual-phase model. Ideally, the stabilized soil forms a skeleton that transfers compressive stress to the EPS beads under pressure, with the EPS beads bearing the majority of the compressive stress. However, when the EPS content is too high, the amount of stabilized soil between the beads becomes insufficient to form an effective skeleton. This lack of structural support prevents adequate stress transfer during deformation coordination between EPS beads and the soil skeleton, leading to specimen failure. Furthermore, as the EPS content increases, more large cracks are observed within the composite stabilized lightweight soil, resulting in a weaker soil structure. These excessive cracks hinder efficient and complete stress transfer during loading, thereby reducing the overall strength of the soil.

The SEM image of lightweight soil stabilized with the cement–fly ash composite curing agent is shown in Figure 9c. After the hydration reaction of cement and fly ash, as well as the secondary hydration reaction between fly ash and cement hydration products, the needle-shaped crystals and large crystal aggregates within the primary cracks were significantly reduced. Additionally, large cracks in the stabilized soil were effectively minimized, resulting in a denser structure and higher soil strength.

Figure 9d illustrates that the hydration reaction between fly ash and cement produces a large amount of flocculent calcium silicate hydrate (C-S-H) gel. This dense gel has no apparent pores or cracks and can fill voids in the soil structure while acting as a binder. This effectively enhances the soil’s strength by improving its structural integrity.

### 3.5. Particle (Pore) and Crack Analysis System (PCAS) Image Processing

To visually analyze the similarities and differences in SEM microimages of lightweight soil stabilized with cement-only and cement–fly ash composite curing agents, as well as to quantitatively evaluate the micro-pore characteristics of the soil, the PCAS system, developed by Dr. Chun Liu’s team from the School of Earth Sciences and Engineering, Nanjing University [25], was used. This system was employed to quantitatively process the number, distribution, and size of pores in lightweight soils treated with different curing agents.

Traditional SEM pore analysis software requires manual identification of pores, manual adjustment of image contrast and grayscale, and manual marking of pores after image adjustment. This method is prone to significant errors, as many small pores may be overlooked during marking, and errors in pore size measurements based on the scale bar are common. The PCAS (Version: 2.324) software, on the other hand, can automatically recognize soil structures and pores, adjust the image contrast and grayscale to appropriate levels, and automatically identify various pores and cracks in the images, providing a range of geometric and statistical parameters. Compared to traditional manual measurement methods, the PCAS system is simpler, more efficient, and ensures repeatability.

**Figure 10:**
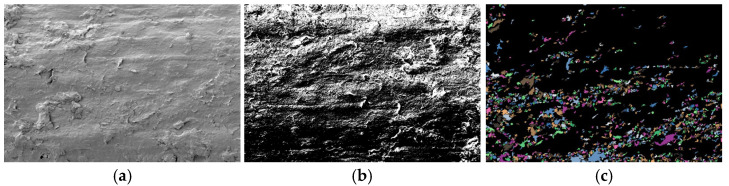
SEM Image Processing of Composite Stabilized Lightweight Soil: (**a**) SEM image, (**b**) binarization, (**c**) vectorization.

**Figure 11:**
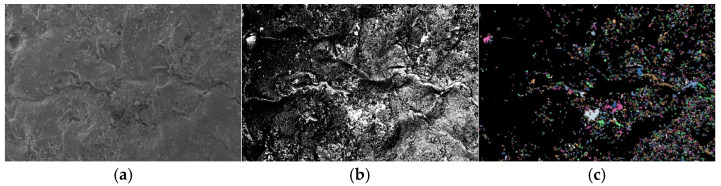
SEM Image Processing of Cement Stabilized Lightweight Soil: (**a**) SEM image, (**b**) binarization, (**c**) vectorization.

Using the PCAS software, binary processing was applied to the SEM microimages of lightweight soil stabilized with cement-only and cement–fly ash composite curing agents. After binary processing, the pore size and distribution characteristics of the SEM microimages became clearly visible, as shown in Figure 10b and Figure 11b.

Figure 10c and Figure 11c present the results of vectorization processing performed on the binary SEM microimages using the PCAS software. After vectorization, the irregular colored geometric shapes in the SEM images represent the dyed pores. A comparison of Figure 10c and Figure 11c reveals that lightweight soil samples stabilized with cement-only exhibit larger pores, and the pore distribution is uneven. In contrast, lightweight soil samples treated with the cement–fly ash composite curing agent show a significant reduction in the number of large pores, with small pore spaces distributed more densely and uniformly. This indicates that the microstructure of soil stabilized with the cement–fly ash composite curing agent differs from that of soil treated with cement only. The former demonstrates a notable decrease in large pores, an increase in the number of small pores, more uniform pore sizes, and a denser soil structure.

Table 4 provides the pore size distribution data obtained from vectorization processing of the SEM microimages using the PCAS software. This table quantitatively and visually displays the distribution of micropores (pore diameter < 10 μm), mesopores (10 μm < pore diameter < 30 μm), and macropores (pore diameter > 30 μm), as well as their respective surface porosity ratios.

The data in Table 4 clearly reveal the pore distribution characteristics of lightweight soil. Most pore diameters in the soil are concentrated between 10–30 µm, with relatively few macropores (pore diameter > 30 μm), and the pores are predominantly small and medium in size. For 10 μm < pore diameter < 30 μm, the number of pores in cement-stabilized lightweight soil is 553, with a surface porosity ratio of 1.762%. For cement–fly ash composite stabilized lightweight soil, the number of pores increases to 711, with a surface porosity ratio of 2.091%, reflecting a 22.23% increase in pore count and a 0.329% increase in surface porosity ratio. For 10 μm < pore diameter < 30 μm, the number of pores in cement-stabilized soil is 1286, with a surface porosity ratio of 12.443%. In the composite stabilized lightweight soil, the pore count decreases to 1060, with a surface porosity ratio of 10.496%, representing a 17.57% reduction in pore count and a 1.947% decrease in surface porosity ratio. For pore diameter > 30 μm, the number of pores in cement-stabilized soil is 72, with a surface porosity ratio of 9.979%. In the composite stabilized lightweight soil, the pore count drops to 44, with a surface porosity ratio of 4.912%, showing a 38.89% reduction in pore count and a 5.066% decrease in surface porosity ratio.

These figures and observations demonstrate that the microstructure of lightweight soil changes significantly after treatment with the composite curing agent compared to cement-only curing. The total number of pores and surface porosity ratio are significantly reduced. This implies that the contact between soil particles and between soil particles and EPS beads is more adequate, which facilitates the formation of stress transfer paths and enhances the skeleton effect of the stabilized soil.

### 3.6. Analysis of Composite Stabilization Mechanism

Based on XRD and SEM tests and previous studies [26], it is evident that the active acidic oxides in fly ash can react with calcium hydroxide crystals produced by cement hydration in a secondary hydration process. This reaction is the primary reason fly ash effectively enhances the strength of cement-based materials:

Fly ash itself undergoes hydration to generate calcium silicate hydrate (C-S-H), which contributes to strength improvement.

The secondary hydration reaction consumes calcium hydroxide crystals and simultaneously generates Type I or Type II calcium silicate hydrate gel. This gel fills the structural voids, resulting in a denser structure (Equation (3)).

The consumption of calcium hydroxide crystals significantly reduces the formation of expansive ettringite crystals, breaking their directional alignment and agglomeration, thereby eliminating the primary cracks in the soil (Equation (4)).

(3) $${Ca(OH)}_{2}+{SiO}_{2}+H_{2}O\to C\text{-}S\text{-}H$$

(4) $${Ca(OH)}_{2}+{Al}_{2}O_{3}+{CaSO}_{4}+H_{2}O\to 3CaO\cdot {Al}_{2}O_{3}\cdot 3{CaSO}_{4}\cdot 32H_{2}O$$

**Figure 12:**
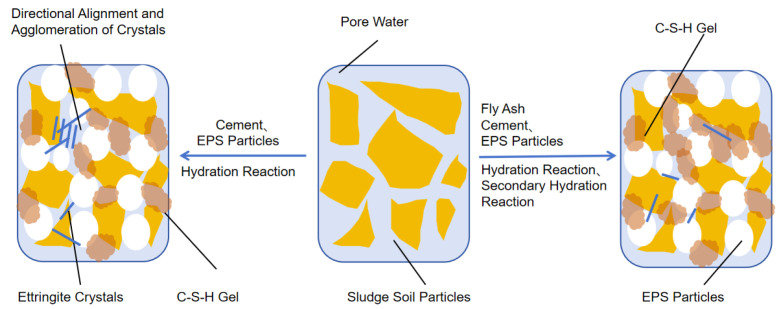
Schematic Diagram of Composite Stabilization Mechanism.

However, the secondary hydration reaction of fly ash depends on the calcium hydroxide crystals produced during the cement hydration process. Thus, when the fly ash content is low, the unconfined compressive strength (UCS) of composite stabilized lightweight soil increases as fly ash is added. Conversely, when the fly ash content is high, there is insufficient cement to generate the calcium hydroxide crystals required for the secondary hydration reaction. Since the strength-enhancing capacity of fly ash hydration is considerably lower than that of Portland cement, the UCS of composite stabilized lightweight soil decreases with further additions of fly ash. And in an alkaline environment, Na^+^ and K^+^ ions in soil particles dissolve and undergo adsorption–exchange with Ca^2+^ (Figure 12).

This ion exchange process is similar to the alkali activation principle in the field of concrete [27]. This process leads to a thinning of the double electric layer of soil particles and a reduction in the interparticle spacing. Smaller soil particles flocculate and aggregate into larger clusters, increasing the cohesion between soil particles.

## 4. Conclusions

In response to strategies such as “sustainable development” and “recycling of resources”, lightweight treatment of dredged sludge from the Yangtze River Basin in Wuhan was conducted using fly ash, cement, and EPS particles. This approach effectively reduces the issues of high costs, resource waste, and environmental pollution associated with large-scale sludge treatment. The main conclusions are as follows:

(1) The composite solidified lightweight soil made from dredged sludge exhibits the characteristics of being lightweight and high strength. Its density, stress–strain behavior, and unconfined compressive strength can be adjusted within a certain range to meet specific engineering needs.
(2) The optimal lightweight treatment for sludge is achieved with 7.5% cement content, 80% EPS content, and 5% fly ash content. Under these conditions, the density of the composite lightweight soil is 1.04 g/cm^3^, which is reduced by 28.27% compared to the original sludge soil density of 1.45 g/cm^3^. The unconfined compressive strength is increased from 110 kPa for the original sludge soil to 551 kPa, effectively improving strength while reducing costs, and meeting the requirements of various backfill soil construction standards.
(3) The use of a cement–fly ash composite stabilizer instead of a traditional single-cement stabilizer results in higher unconfined compressive strength for lightweight sludge soil. This is because fly ash reacts with calcium hydroxide crystals, an intermediate product of cement hydration, to undergo secondary hydration reactions, which reduce the formation of expansive ettringite while generating a more hydrated C-S-H gel. Quantitative analysis revealed that the microstructure of the lightweight soil treated with the composite stabilizer changed significantly, with a noticeable reduction in pore number and porosity. This indicates more sufficient contact between soil particles and between soil particles and EPS particles, which facilitates the skeleton formation and stress transmission function of solidified soil particles.

This study can provide a reference for the treatment of dredged sludge in the Yangtze River Basin of Central China. At this stage, the density and unconfined compressive strength of the composite solidified lightweight soil made from dredged sludge have met the standard requirements. Future research will focus on its durability, conducting tests such as dry–wet cycles, freeze–thaw cycles, cyclic loading, and dynamic loading to determine the service life of the material and make continuous improvements.

## Figures and Tables

**Table 1:** Basic Physical Parameters of Dredged Sludge.

Natural Water Content/%	Liquid Limit/%	Plastic Limit/%	Liquidity Index	Plasticity Index	Optimum Water Content/%	Maximum Dry Density/g/cm^3^
96.2	63.5	30.2	2.88	33.3	33	1.089

**Table 2:** Main Chemical Components of Fly Ash.

**Components**	Al_2_O_3_	SiO_2_	SO_3_	CaO	Free CaO	MgO	Fe_2_O_3_
**Content/%**	24.2	45.1	2.1	5.6	0.9	2.4	5.2

**Table 3:** Test Scheme.

EPS Content/%	Cement Content/%	Curing Period (Days)
60	7.5	0, 2.5, 7.5, 10
80		0, 2.5, 7.5, 10
100		0, 2.5, 7.5, 10

**Table 4:** Pore Distribution Table.

Pore Diameter (d)/µm	Cement Stabilized Lightweight Soil		Cement–Fly Ash Composite Stabilized Lightweight Soil	
	Pore Count	Surface Porosity/%	Pore Count	Surface Porosity/%
<10	553	1.762	711	2.091
10–30	1286	12.443	1060	10.496
>30	72	9.979	44	4.912

## Data Availability

All data generated or analyzed during this study are included in this published article.
